# Early stages of aggregation of engineered α-synuclein monomers and oligomers in solution

**DOI:** 10.1038/s41598-018-37584-6

**Published:** 2019-02-11

**Authors:** Xi Li, Chunhua Dong, Marion Hoffmann, Craig R. Garen, Leonardo M. Cortez, Nils O. Petersen, Michael T. Woodside

**Affiliations:** 1 Department of Chemistry, University of Alberta, Edmonton, AB T6G 2G2 Canada; 2 Department of Physics, University of Alberta, Edmonton, AB T6G 2E1 Canada; 3 Division of Neurology, Department of Medicine, Centre for Prions and Protein Folding Diseases, and Neuroscience and Mental Health Institute, University of Alberta, Edmonton, AB T6G 2M8 Canada

## Abstract

α-Synuclein is a protein that aggregates as amyloid fibrils in the brains of patients with Parkinson’s disease and dementia with Lewy bodies. Small oligomers of α-synuclein are neurotoxic and are thought to be closely associated with disease. Whereas α-synuclein fibrillization and fibril morphologies have been studied extensively with various methods, the earliest stages of aggregation and the properties of oligomeric intermediates are less well understood because few methods are able to detect and characterize early-stage aggregates. We used fluorescence spectroscopy to investigate the early stages of aggregation by studying pairwise interactions between α-synuclein monomers, as well as between engineered tandem oligomers of various sizes (dimers, tetramers, and octamers). The hydrodynamic radii of these engineered α-synuclein species were first determined by fluorescence correlation spectroscopy and dynamic light scattering. The rate of pairwise aggregation between different species was then monitored using dual-color fluorescence cross-correlation spectroscopy, measuring the extent of association between species labelled with different dyes at various time points during the early aggregation process. The aggregation rate and extent increased with tandem oligomer size. Self-association of the tandem oligomers was found to be the preferred pathway to form larger aggregates: interactions between oligomers occurred faster and to a greater extent than interactions between oligomers and monomers, indicating that the oligomers were not as efficient in seeding further aggregation by addition of monomers. These results suggest that oligomer-oligomer interactions may play an important role in driving aggregation during its early stages.

## Introduction

α-Synuclein is a small, intrinsically disordered protein^1^ that is abundant in neurons and localized primarily to presynaptic terminals^2,3^. While its precise cellular functions remain unclear, α-synuclein has been implicated in protecting neurons from apoptotic stimuli^4^ and in various aspects of synaptic vesicle trafficking^5–7^. It is a target of considerable interest because of its association with Parkinson’s disease (PD) and related neurodegenerative disorders. Lewy bodies, cytoplasmic inclusions composed mainly of amyloid fibrils of α-synuclein^8^, are a prominent clinical feature of PD, dementia with Lewy bodies^9^, and multiple system atrophy^10^, suggesting that α-synuclein aggregation contributes to neuronal degeneration and the clinical manifestations of these diseases. Furthermore, multiple single-point mutations of the gene encoding α-synuclein are linked to familial forms of Parkinson’s disease^11–16^, as are gene multiplications that can lead to its over-expression^17^. Several of these mutations accelerate the aggregation of α-synuclein^18^, heightening suspicion that α-synuclein contributes to disease progression.

α-Synuclein aggregation has been studied extensively in the last two decades. A number of conditions have been found to facilitate α-synuclein aggregation *in vitro*, including low pH (2.0–5.5)^19^; the presence of organic solvents^20^; elevated temperature (37–57 °C)^19^; high α-synuclein concentration (>8 mg/mL)^21^; the presence of small, pre-formed α-synuclein fibrils (‘seeds’)^21,22^; the presence of multivalent metal ions like Al(III), Fe(III), Cu(II), and Co(III)^21,23,24^; the presence of lipids or membranes^25,26^; and specific mutations^15,27^. Like many other misfolding proteins exhibiting sigmoidal amyloid growth kinetics^28,29^, α-synuclein aggregation appears to involve a nucleated self-assembly process that ultimately leads to fibril formation^30–32^. The aggregation starts with a lag phase characterized by a very slow growth rate, during which monomers co-exist with transient small oligomers, followed by a dramatically increased growth rate of β-structured fibrillar material^33^. The small oligomers formed during the lag phase have been termed critical nuclei, because their formation is the rate-limiting step for the aggregation process^34^. Furthermore, it is these oligomeric species that are thought to be the most toxic entities leading to disease^35–37^.

Various methods have been applied previously to study α-synuclein aggregation. For example, electron microscopy and X-ray diffraction techniques have been used to provide information on morphology of amyloid fibrils^38–40^. Thioflavin T (ThT) fluorescence^41,42^, meanwhile, has been used to measure the kinetics of fibril formation^43,44^. Although these approaches are sensitive to the formation of fibrillar structures, they reveal less about earlier aggregation stages, namely nucleation and oligomerization, which can themselves be very complex. Single-molecule (SM) methods are particularly well-suited to studying these early stages^45–47^, and a variety of SM techniques have been used to study the interactions between α-synuclein molecules leading to aggregation, including fluorescence resonance energy transfer (FRET)^48–52^, fluorescence correlation spectroscopy (FCS)^48,53,54^, and force spectroscopy^55–57^. Such studies have suggested that early-stage oligomers play a key role as nuclei in the aggregation process. However, the heterogeneity of oligomers and their transitory nature^58^ make it difficult to study their role in the α-synuclein aggregation process.

To address these challenges, we engineered tandem-repeat oligomers of α-synuclein having a specified size by connecting two, four, or eight monomers head-to-tail with a three-amino-acid linker between each repeated domain^55,56,59^. This approach, which has been previously applied to study aggregation in other proteins^60–62^, allowed us to study the properties of oligomers of different sizes in a controlled way. Using these engineered oligomers, we probed size-dependent pairwise intermolecular interactions during α-synuclein aggregation, applying a modified FCS assay that allowed us to characterize early oligomers containing two fluorophores (dual-color fluorescence cross-correlation spectroscopy, FCCS)^63^. Dual-color FCCS is superior to standard FCS for studying the dynamics of aggregation because it ensures that particles labelled with two colors must represent aggregates^63^, it allows sensitive discrimination between small differences in molecular weight between two species^64^, and the dual-color labelling allows aggregates formed from different proteins to be detected. Measuring the extent of association between oligomeric species of different sizes at various points during the earliest stages of aggregation, we found that the tandem oligomers preferentially self-associated rather than acting as seeds to accelerate the addition of monomers.

## Results

### FCS indicates monomers and engineered oligomers are disordered in solution

**Figure 1:**
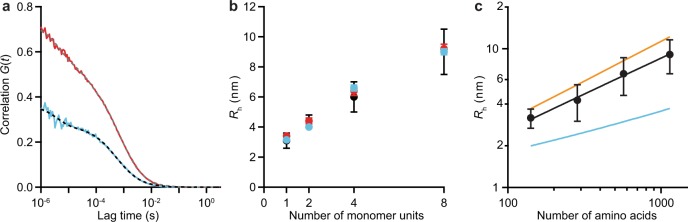
FCS of α-synuclein constructs. (**a**) Auto-correlation curves for dye-labelled αS-8 (red: Cy5-labelled, cyan: Oregon green-labelled. (**b**) *R*_h_ values for each α-synuclein construct agree whether determined from FCS (red: Cy5-labelled constructs, cyan: Oregon green-labelled constructs) or DLS (black). Error bars for FCS are close to the size of the data symbols. (**c**) *R*_h_ for α-synuclein constructs (black) shows a power-law dependence on the number of amino acids, closer to the size-dependence of *R*_h_ predicted for a fully denatured polypeptide chain (orange) than for a folded globular protein (cyan). All error bars represent s.e.m.

Samples of α-synuclein monomers (αS-1), tandem dimers (αS-2), tandem tetramers (αS-4), and tandem octamers (αS-8) were expressed, purified, and labelled fluorescently at the C terminus with either green or red dyes as described in the Methods section. Before studying inter-molecular interactions using FCCS, we first measured the behavior of each construct using standard FCS (Fig. S1). The auto-correlation curves of the fluorescence signal from the red-labelled and green-labelled versions of each construct, measured in separate experiments, were fit to Eqs 1 and 2 (see Methods) to determine the diffusion time for each construct (Fig. 1a). To ensure that the fluorescence detected was independent of α-synuclein auto-fluorescence, we repeated the experiments with constant amounts of labelled construct and increasing amounts of unlabelled construct. We found that although the unlabelled α-synuclein contributed slightly to fluorescence, the effect was small (Table S1), suggesting that auto-fluorescence at the wavelengths used could be ignored as it did not interfere with the measurements.

The hydrodynamic radius (*R*_h_) corresponding to the measured diffusion time for each construct was calculated from Eq. 3 (Fig. 1b). Previous work^59^ had measured *R*_h_ of unlabelled αS-1, αS-2, αS-4, and αS-8 using both size exclusion chromatography^65^ and dynamic light scattering (DLS)^66,67^ by comparing each protein construct to native or denatured globular protein standards. The results found from FCS (Fig. 1b, red and cyan) were very similar to those found from DLS (Fig. 1b, black): we observed a monotonic increase in *R*_h_ with the size of the oligomer. The *R*_h_ calculated for the monomer, 3.27 ± 0.04 nm (all errors reported as standard error of the mean), also agreed well with previous results^19^. The labeling with Oregon green 488 or Cy5 fluorophores thus did not materially affect *R*_h_.

The hydrodynamic radius of a protein can be predicted based on the number of amino acids in the protein, *N*: *R*_h_ = 0.475 × *N* ^0.29^ nm is expected for a natively folded (globular) protein, whereas *R*_h_ = 0.221 × *N*^0.57^ nm is expected for a fully denatured protein^68^. Comparing the FCS results to these predictions for globular and unstructured proteins (Fig. 1c, cyan and orange, respectively), all four protein constructs (Fig. 1c, black) were found to fall between the two predicted limits, but closer to the unstructured limit. This observation is consistent with previous work showing that monomeric α-synuclein is largely disordered *in vitro* at neutral pH^19,59^, as are the engineered oligomers^59^, but that both monomeric α-synuclein^69,70^ and tandem oligomers^55,56^ are somewhat more compact than a purely random coil. Quantifying the correlation between *R*_h_ and *N* for the tandem α-synuclein constructs, we found *R*_h_ = (0.25 ± 0.03) × *N* ^0.51±0.02^. This result suggests that there is no significant difference in the degree of compactness between monomers and tandem oligomers in solution, and it may thus be used to estimate the effective size of small aggregates of our constructs under the assumption that they retain a similar level of compactness.

### FCCS indicates tandem oligomers self-aggregate to a greater extent than monomers

**Figure 2:**
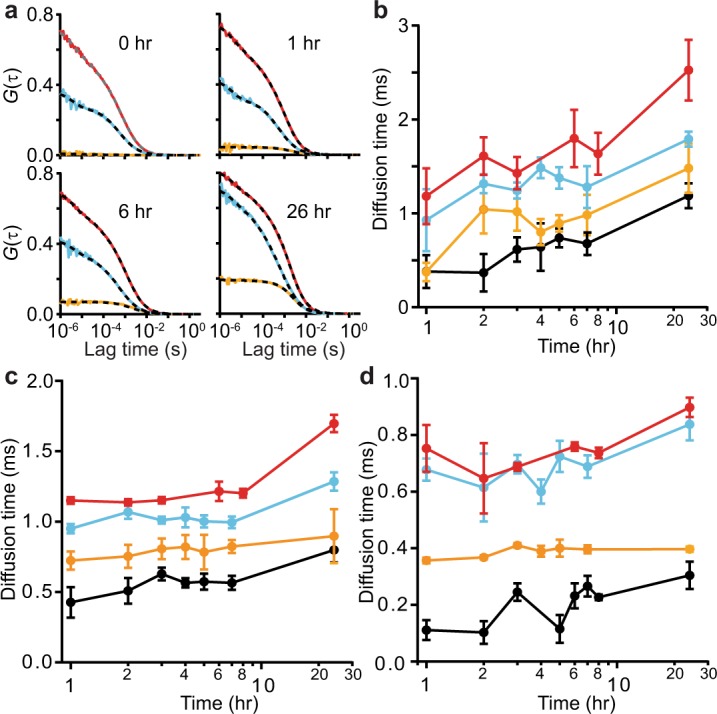
Self-aggregation of different α-synuclein constructs observed by FCCS. (**a**) Representative auto-correlation (red: Cy5-labelled, cyan: Oregon green-labelled); and cross-correlation (orange) curves for αS-8 at different incubation times. (**b**) Average diffusion time of dual-labelled aggregates for self-aggregation of αS-1 (black), αS-2 (orange), αS-4 (cyan), and αS-8 (red) at different incubation times. (**c**) Diffusion time of single-color aggregates labelled with Cy5. (**d**) Diffusion time of single-color aggregates labelled with Oregon green. Note that the non-monotonic fluctuations in diffusion time reflect the statistical variability and are not significant. Error bars represent s.e.m from 2–5 replicates of 50 measurements each.

To measure the association of protein molecules as they formed aggregates, one construct (αS-1, -2, -4, or -8) labelled with Oregon green 488 was incubated with another construct (αS-1, -2, -4, or -8) labelled with Cy5 for times varying from 1–24 hr, and the correlations of the fluctuations in the two fluorescence signals—auto-correlations for each color as well as cross-correlation between colors—were monitored (Fig. S2). Sample correlation curves for αS-8 are presented as a function of incubation time in Fig. 2a. Auto-correlation curves were fit as for the FCS measurements, and cross-correlation curves representing the behavior of dual-colored aggregates were fit with Eq. 4. Correlations were initially fit with multiple diffusion times to reflect the heterogeneous mixture of species expected during the early stages of aggregation^32^. However, increasing the number of species used in the calculation failed to improve the fitting when compared to fits using a single diffusion time. Ultimately, the best estimates of the mixture of species in the confocal volume were achieved by fitting the correlations under the assumption of a single diffusion time, but allowing the coefficient α (representing anomalous diffusion) to vary, as shown by application of the Bayesian information criterion (ΔBIC ~ 15)^71^.

The cross-correlation results from the dual color self-aggregation experiments (*i*.*e*. monomer with monomer, (αS-1)_r_(αS-1)_g_; dimer with dimer, (αS-2)_r_(αS-2)_g_; tetramer with tetramer, (αS-4)_r_(αS-4)_g_; and octamer with octamer, (αS-8)_r_(αS-8)_g_) revealed that diffusion times increased as a function of incubation time. This outcome, indicating the formation of larger aggregates over time, occurred for each of the tandem oligomers (Fig. 2b). Looking instead at the auto-correlation signals to monitor the growth of single-color self-aggregates for Cy5-labelled (Fig. 2c) and Oregon green-labelled (Fig. 2d) constructs, we found that the average diffusion time increased more slowly than for the dual-color aggregates, particularly in the first 8 hours. This difference likely reflects the fact that the auto-correlations are heavily biased by the signal from the un-aggregated molecules that predominate early in the incubation. In contrast, the cross-correlation signal is sensitive exclusively to aggregates, because it only detects assemblies in which at least one molecule with each color of dye is present. An added advantage of the FCCS assay is that, because the proteins with different dye labels are kept apart until the start of the incubation, there can be no pre-formed dual-colored seeds.

### FCS and FCCS measurements occur during the lag phase of aggregation

**Figure 3:**
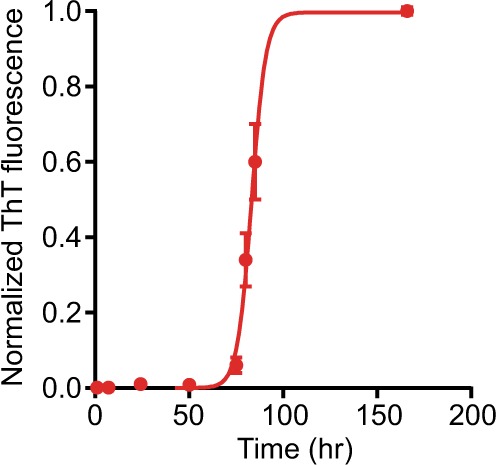
Fibrillisation of αS-1 by ThT. Kinetics of αS-1 fibrillization were monitored using ThT fluorescence, under unseeded conditions identical to those used for FCCS measurements. Error bars represent s.e.m.

To ensure that the FCS and FCCS measurements reflected events during the early stages of aggregation (the lag phase), we repeated the aggregation measurements while monitoring the fluorescence emission of Thioflavin T (ThT) at 490 nm. ThT fluorescence probes the formation of fibrillar structures, but is insensitive to precursor oligomers^72^. ThT fluorescence from αS-1 measured under conditions exactly duplicating those in the FCCS experiments showed a lag time of ~60 h (Fig. 3), indicating that the formation of mature fibrils was slow even at the relatively high protein concentration used, owing to the lack of pre-formed seeds, beads, and other such factors that help accelerate fibrillization^73–75^. Notably, the lag time of ~2.5 days indicates that the FCCS measurements were all completed while still in the early phases of aggregation, well before the appearance of mature fibrils. ThT measurements of the aggregation of the tandem oligomers were also done, using a microplate reader (Fig. S3). Lag times were again much longer than the incubation times used in FCCS assays, indicating that the FCCS results reflect the earliest stages of aggregation: the lag time was ~100 h for αS-2, ~150 h for αS-4, and >200 hr for αS-8. Note that although fibrillization of αS-8 was not observed in this assay, αS-8 does indeed form fibrils, as seen in previous work using electron microscopy to verify that the fibrils formed by the tandem oligomers were morphologically similar to those formed by monomeric α-synuclein^59^.

### Larger oligomers incorporate into aggregates more quickly than monomers in self-aggregation experiments

The average number of labelled proteins or aggregates observed in the confocal volume was related to the amplitude of the corresponding correlation function^76^. Whereas the amplitudes of the auto-correlation functions, *G*_r_(0) and *G*_g_(0), fluctuated a little over time (Fig. 2c,d), likely owing to the dynamic population of aggregating molecules present (and to a lesser extent to variations arising from dilution), the amplitude of the cross-correlation function, *G*_rg_(0), increased systematically with incubation time, indicating that the tandem oligomers labelled with different fluorophores interacted to form dual-labelled aggregates (Fig. 2b). To track the growth of the aggregates more quantitatively, we re-expressed the results in terms of the density of the fluorescence-emitting particles: *n*_*r*_, *n*_*g*_, and *n*_*rg*_ for red, green, and dual-color aggregates, respectively. These densities were calculated by normalizing the number of particles observed by the effective confocal volumes (respectively *V*_*r*_ = 1.43 fL, *V*_*g*_ = 1.29 fL, and *V*_*rg*_ = 1.15 fL), for each construct used in the dual-color FCCS self-aggregation measurements: (αS-1)_r_(αS-1)_g_, (αS-2)_r_(αS-2)_g_, (αS-4)_r_(αS-4)_g_, and (αS-8)_r_(αS-8)_g_.

**Figure 4:**
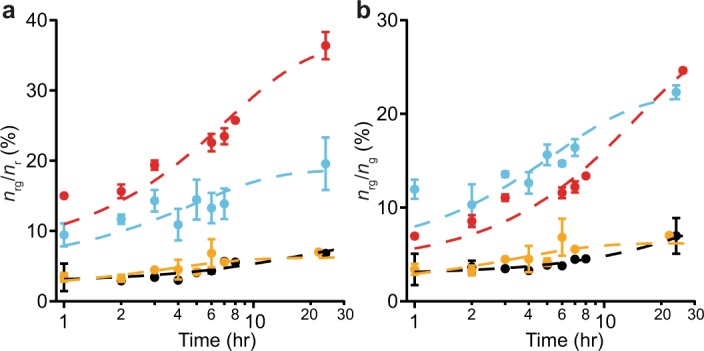
Self-aggregation kinetics of α-synuclein constructs. (**a**) Time course of the average fraction of Cy5-labelled α-synuclein constructs incorporated into dual-colored aggregates. (**b**) Time course of the average fraction of Oregon green-labelled constructs incorporated into dual-colored aggregates. Dashed lines indicate fit to single-exponential growth. Black: αS-1, orange: αS-2, cyan: αS-4, red: αS-8. Error bars represent s.e.m.

During self-aggregation, the density of aggregates emitting both colors increased systematically with incubation time (Fig. 4). The incorporation dynamics of red-labelled (Fig. 4a) and green-labelled α-synuclein (Fig. 4b) were quantified via the time evolution of the ratio of dual-color aggregates to single-color species. After 24 hr, ~35% of Cy5-labelled αS-8 and ~15% of Cy5-labelled αS-4 was incorporated into dual-color aggregates, as opposed to only ~5% of αS-1 and αS-2 (Fig. 4a), indicating faster aggregation for the larger constructs under these conditions. The same general trends were seen for incorporation of Oregon green-labelled α-synuclein into the dual-colored aggregates (Fig. 4b). Although the full kinetics of aggregation are complex^77^, those of the early stages may be reasonably well described by simple exponential growth^32,78^. Fitting the time course of aggregation for each construct to single exponentials (Fig. 4, dashed lines), we found that the rate of aggregation of αS-1 (~ 0.04 hr^−1^) was consistent with previous results for similar monomeric α-synuclein constructs^32,48^ but considerably smaller than the corresponding rates for the tandem oligomers (Table 1). Furthermore, the extent of aggregation also tended to be greater for larger oligomers.

**Table 1:** Exponential growth fitting parameters for self–aggregation experiments.

	*n*_rg_/*n*_r_		*n*_rg_/*n*_g_	
	|*A*| (%)	*k* (h^−1^)	|*A*| (%)	*k* (h^−1^)
(αS-1)_r_(αS-1)_g_	6 ± 1	0.043 ± 0.003	8 ± 2	0.035 ± 0.005
(αS-2)_r_(αS-2)_g_	5 ± 1	0.23 ± 0.03	4 ± 1	0.20 ± 0.03
(αS-4)_r_(αS-4)_g_	14 ± 3	0.17 ± 0.03	17 ± 2	0.18 ± 0.03
(αS-8)_r_(αS-8)_g_	29 ± 3	0.15 ± 0.03	26 ± 3	0.06 ± 0.01

### Tandem oligomers are not efficient nucleation sites for monomer addition

**Figure 5:**
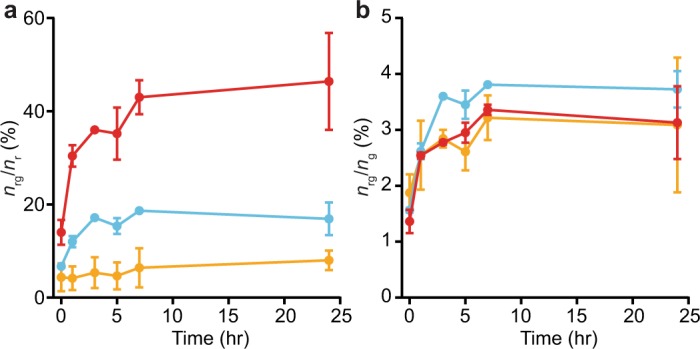
Monomer-oligomer co-aggregation kinetics. (**a**) Fraction of Cy5-labelled oligomers (orange: αS-2, cyan: αS-4, red: αS-8) incorporated into dual-colored co-aggregates with Oregon green-labelled monomers. (**b**) Fraction of Oregon green-labelled monomers incorporated into dual-colored co-aggregates with oligomers (orange: αS-2, cyan: αS-4, red: αS-8). Error bars represent s.e.m.

To test whether the tandem oligomers can act as nucleation sites for monomer addition, we mixed red-labelled oligomers with green-labelled monomers—(αS-2)_r_(αS-1)_g_, (αS-4)_r_(αS-1)_g_, and (αS-8)_r_(αS-1)_g_—and measured the incorporation of green-labelled protein into dual-color aggregates (Fig. 5). The tandem oligomers still tended to self-associate to form larger aggregates (Fig. 5a), but only ~2% of monomers were involved in these aggregates (Fig. 5b), indicating very little incorporation of monomers. The discrepancy between the extent of self-aggregation of the tandem oligomer versus co-aggregation with the monomer grew larger with oligomer size (Table 2). Repeating the experiments by swapping the monomer and oligomer fluorophores yielded qualitatively similar results (Table 2), with oligomers remaining more likely to add to aggregates than monomers. These results suggest that the tandem oligomers are at best poor for seeding monomer addition to aggregates, even with a molar excess of monomers. The picture that emerges is that small co-aggregates form fairly rapidly, closer to the rate of formation of oligomer self-aggregates than monomer self-aggregates. Once small co-aggregates have formed, however, the addition of monomers rapidly tapers off and oligomer-oligomer interactions are instead the preferred mechanism for continued growth.

**Table 2:** Exponential-growth fitting parameters for co-aggregation experiments.

	*n*_rg_/*n*_r_		*n*_rg_/*n*_g_	
	|*A*| (%)	*k* (h^−1^)	|*A*| (%)	*k* (h^−1^)
(αS-2)_r_(αS-1)_g_	4 ± 1	0.11 ± 0.08	1.7 ± 0.4	0.4 ± 0.3
(αS-4)_r_(αS-1)_g_	11 ± 1	0.23 ± 0.04	2 ± 0.1	0.8 ± 0.4
(αS-8)_r_(αS-1)_g_	40 ± 7	0.12 ± 0.05	1 ± 0.2	0.4 ± 0.2
(αS-1)_r_(αS-2)_g_	5 ± 1	0.2 ± 0.1	4 ± 1	0.2 ± 0.1
(αS-1)_r_(αS-4)_g_	1.0 ± 0.2	2 ± 1	20 ± 3	0.5 ± 0.2
(αS-1)_r_(αS-8)_g_	4 ± 2	0.05 ± 0.04	11 ± 3	0.2 ± 0.2

### Zeta potentials allow for diffusion-limited aggregation of the α-synuclein constructs

The zeta potentials of the α-synuclein constructs dispersed with PBS were calculated from DLS measurements and found to be −9.1 ± 0.3 mV for αS-1, −8.3 ± 0.8 mV for αS-2, −8.5 ± 0.3 mV for αS-4, and −11.4 ± 0.3 mV for αS-8, respectively. Calculating the surface charge density of each α-synuclein construct at its slipping plane from the zeta potential^79^, we found a charge of −6 for αS-1, −10 for αS-2, −21 for αS-4, and −55 for αS-8. These results are close to the expectation that each α-synuclein monomer unit at neutral pH has a net of ~8 negatively charged amino acid residues^80^, but ~30% smaller, possibly due to shielding by counter-ions in the Stern layer^81^.

**Figure 6:**
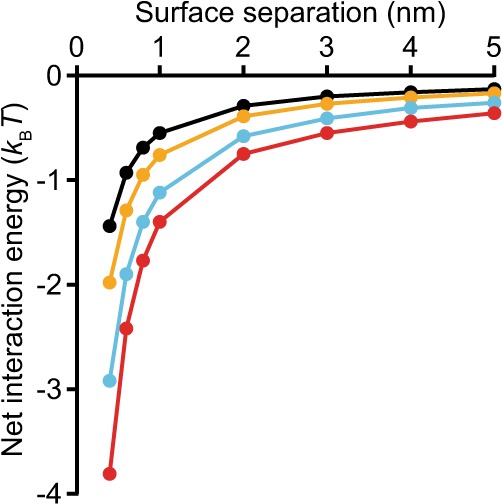
Net interaction energy of α-synuclein constructs. The total interaction potential between two charged surfaces of a given α-synuclein construct in aqueous electrolyte solutions with the corresponding zeta potential is shown (black: αS-1, orange: αS-2, cyan: αS-4, red: αS-8).

Assuming that DLVO theory can be applied to these small protein particles^82^, we estimated the total potential energy of interaction between α-synuclein species. We found that the net energy of interaction between two protein particles was always negative, at all separations (Fig. 6). This result suggests that electrostatic repulsion between the highly-charged protein molecules is not sufficient to prevent the aggregation^83^, which therefore should be diffusion-controlled with a rate constant on the order of 10^9^ M^−1^ s^−1^. However, the initial rates observed here and elsewhere^32,48^ are much slower than the diffusion-limited rate, consistent with the notion that other factors control the aggregation process.

## Discussion

Aggregation is a complex process involving many steps, including primary nucleation (formation of an initial oligomer), oligomer growth, structural reconfiguration of oligomers, protofibril formation and fibril maturation, fragmentation of large oligomers and fibrils, and secondary nucleation^33^. The work here focused on the initial stages of the process: primary nucleation and oligomer growth. Considering first the nucleation of aggregates, we note that the absolute values of the zeta potentials are in the range where inter-particle repulsion is not significant^84^, hence such repulsion should not generate any barrier to spontaneous nucleation from two-body collisions. α-Synuclein is physiologically abundant in presynaptic neuron termini^2,3^, hence it ought collide with some frequency. The observations that few of these encounters result in nucleation of aggregates^33,50^ and that nucleation is a slow process with apparently large thermodynamic barriers^22^ suggest that nucleation involves additional pre-requisites, such as a specific relative orientation of molecules, conformational reorganization, and/or specific conditions or additional factors (ionic strength, pH, membrane surfaces,…)^85–88^.

Comparing the aggregation of the tandem oligomers to the monomer, we found that the extent of aggregation increased with oligomer size, and the tandem oligomers aggregated at a faster rate than the monomers. Accounting for differences in molecular concentration to make quantitative comparisons between the rates for self-aggregation of the different constructs, the rate for the dimer was ~10.5-fold higher than for the monomer, that for the tetramer was ~17-fold higher, and that for the octamer was ~13.5-fold higher. Tethering two monomer units together thus led to an order of magnitude increase in aggregation rate, but further increases in the number of monomers tethered together yielded diminishing returns (and an apparent decrease in the rate from tetramer to octamer, although the difference was not statistically robust). This size-dependent difference in the rate of nucleation between the oligomers and monomers cannot be due to differences in surface charge density, as the zeta potentials were effectively the same for all of the constructs studied, but must instead be related to some other property of the oligomers, such as a greater ability to achieve the conformational fluctuations needed to induce nucleation. The enhanced ability of the tandem oligomers to aggregate was also reflected in the co-aggregation measurements probing the ability of the oligomers to stimulate aggregation of monomers: even though there were more monomer molecules present than tandem oligomers, the latter added on to the aggregates to a much greater extent after nucleation. The nucleation rate of monomer-oligomer complexes, however, showed no statistically robust trend with oligomer size when corrected for the different oligomer concentrations (although there was a hint that tetramers might induce faster nucleation than the other oligomers). Oligomer-oligomer interactions thus seemed to dominate over oligomer-monomer or monomer-monomer interactions as a mechanism for aggregate growth, at least at the early stages of aggregation probed here.

Previous work has suggested that various intermediates in the aggregation process can be differentiated not only by their relative neurotoxicity and β-structured content^32,89,90^ but also via the kinetics of each stage: the elongation of relatively disordered nuclei through monomer addition and the continued growth of these aggregates after conversion into more ordered structures occurred at different rates, with the first stage noticeably faster than the second^32^. Monomeric α-synuclein aggregated at a rate consistent with the initial stage observed previously, suggesting that it was forming relatively disordered aggregates (we observed no second phase on the timescale of our measurements). The large difference in aggregation rates between the monomers and tandem oligomers might, by analogy to the previous work, reflect differences in the structures of the aggregates formed, with the aggregates of tandem oligomers being more ordered. The fact that tandem oligomers extend the growth of small aggregates more easily than monomers is consistent with this notion, putatively reflecting a need for the monomers to reconfigure into a more ordered state upon addition to the growing aggregate, which slows down the aggregate growth. However, this interpretation is undercut by previous characterizations of the tandem oligomers showing that they all retain a similar amount of disorder as monomeric α-synuclein^59^. Another possible explanation is that the linkers between monomer domains in the tandem oligomers may accelerate aggregation because the constraints imposed by the linkers reduce the conformational space to be searched (since the linked monomers form only a subset of all conformations possible for oligomers consisting of unrestrained monomers) and/or bias the conformational ensemble occupied by the tandem oligomers to favor conformations amenable to aggregation. Conceivably, the tandem oligomers could also bypass the relatively disordered aggregate phase found in previous work on monomers^32^ and transform directly into a more structured aggregate; this possibility would need to be tested in future studies probing the internal structure of the tandem oligomers within the aggregates.

One apparent contradiction in the FCCS data is that the aggregation rates are faster for the large tandem oligomers than for the monomers, but the diffusion times suggest that the efficiency of the aggregation is lower for the larger tandem oligomers, because the aggregates incorporate a smaller number of molecules. This result may be explained by the different biases inherent in these two analyses. The FCCS amplitude is more sensitive to the larger aggregates, because their fluctuations contribute more to the correlation functions, whereas the diffusion time is more sensitive to the smaller (and hence faster-moving) aggregates, because fitting to a single diffusion time will tend to de-emphasize the effect of the long tail in the correlation function that arises from the larger, slower aggregates. Accordingly, the absolute values of these average parameters must be used with caution whenever there is an underlying distribution of oligomers in solution.

Lastly, we note that according to previous work^59^, the monomer and tandem oligomers all start the aggregation process in similarly disordered states, and they also have very similar endpoints, namely morphologically similar amyloid fibrils. Our current work, however, reveals that despite their aggregation starting and ending in similar states, the monomers and tandem oligomers of different sizes have detectable differences in how they behave in the early stages of the process. A likely explanation is that although the head-to-tail linkages between monomer domains in the tandem oligomers do not prevent amyloid fibrils from forming, they do constrain the possible conformations of the protein during intermediate steps and alter the kinetics or thermodynamics of competing pathways, as mentioned above. Changing the constraints imposed by linkers (*e*.*g*. by changing the linker length, the linkage orientation, or the points at which linkers are attached) so as to bias the possible pathways in particular ways may then offer the opportunity to parse the contributions of different pathways to the different stages of aggregation.

## Methods

### Design, synthesis and purification of α-synuclein constructs

The genes for expressing α-synuclein dimer, tetramer, and octamer constructs were made by linking repeats of the wild-type monomer gene in tandem with a peptide linker as described previously^55,56,59^. The tandem geometry linking the C and N termini of adjacent units was chosen to enable the construction of oligomers containing multiple monomer domains; it is possible to conjugate monomers in different linking geometries, *e*.*g*. linking two C termini or two N termini^62^, but multiplexing such approaches to generate tetramers and octamers is impractical. The linkers were all GSG tri-peptides, similar to linkers used in previous work on aggregation of tandem-repeat proteins^60,61^. To optimize yields, open reading frames were cloned into pET21a expression plasmids (EMD Millipore, Etobicoke, ON, Canada). Each plasmid construct was confirmed by restriction endonuclease digests and DNA sequencing. A cysteine residue was added to the C terminus of each construct via site-directed mutagenesis (Agilent Technologies, Mississauga, ON, Canada) to allow for fluorescent labeling. Each cysteine variant was likewise confirmed by DNA sequencing.

For protein expression, competent Rosetta 2 (DE3) cells (EMD Millipore) were transformed to ampicillin resistance with plasmids for each protein construct. Thereafter, 1 L LB medium supplemented with 100 µg/mL ampicillin (SigmaAldrich, Oakville, ON, Canada) and 34 µg/mL chloramphenicol (SigmaAldrich) was inoculated and grown at 310 K with shaking (225 rpm) until the optical density at 600 nm reached 0.6. Construct over-expression was induced by adding isopropyl β-D-1-thiogalactopyranoside (Gold Biotechnology, Olivette, MO, USA) to a final concentration of 2 mM. Cells were cultivated at 303 K and 225 rpm for an additional 5 hours before harvesting by centrifugation. Similar to methods previously described^59,91^, the expressed α-synuclein constructs were released from the *E*. *coli* periplasm using an osmotic shock procedure. Following ammonium sulfate precipitation of the proteins released from the periplasm, precipitated protein was resuspended and purified on an anion exchange column, eluting with a linear gradient of NaCl. Fractions confirmed to contain pure construct were pooled and the purity of all proteins was tested by SDS-PAGE. The αS-8 construct required additional purification by size exclusion chromatography. The purified product for each construct was precipitated with ammonium sulfate and centrifuged prior to immersion of pellets in liquid nitrogen for storage at 193 K.

C-terminally Cys-tagged α-synuclein constructs were conjugated to maleimide-linked Oregon green 488 dye (ThermoFisher Scientific, Mississauga, ON, Canada) as described^92^, and the labelled protein was purified from excess free dye by buffer exchange using an Amicon ultracentrifugal filter device (ThermoFisher Scientific) with appropriate molecular weight cut-off. For efficient labeling of α-synuclein with the red dye (Cy5), the Cys-tagged constructs were first conjugated with a *trans*-cyclooctene-PEG_3_-maleimide (TPM) linker (Click Chemistry Tools, Scottsdale, AZ, USA). Excess TPM was then removed via centrifugal filtration and the product labelled with an equimolar concentration of tetrazine (Tz)-modified Cy5 dye (Click Chemistry Tools) at 277 K with continuous stirring for 4 hours. Excess Tz-Cy5 dye was removed using an Amicon ultra-centrifugal filter.

### FCS measurements and analysis

FCS measurements were performed using a laser scanning confocal microscopy system (Carl Zeiss LSM 510/ConfoCor 2) equipped with an argon multi-line laser operating at 488 nm and a helium-neon laser at 633 nm. To eliminate fluorescence crosstalk during measurements, laser wavelength cutoffs were established (green 505–540 nm, red 655–710 nm), then lasers were collimated into a water immersion objective (Carl Zeiss C-Apochromat 40x/1.20 W DicIII) and focused onto overlapping focal volumes (Fig. S1). The system was calibrated by measuring the diffusion coefficients of rhodamine 6 G (SigmaAldrich), Oregon green 488 (ThermoFisher Scientific) and Cy5-tetrazine (Click Chemistry Tools) in PBS at 298 K, comparing to established values for the identical conditions (respectively: 4.14 × 10^−6^, 4.11 × 10^−6^, and 3.6 × 10^−6^ cm^2^ s^−1^)^93^. Based on these data-sets, we derived the effective confocal volumes for each of the two colors individually: *V*_r/g_ = (π/2)^3/2^[*w*_*xy*_^2^]_r/g_[*w*_*z*_]_r/g_, as well as for their cross-correlation: *V*_rg_ = (π/2)^3/2^([*w*_*xy*_^2^]_r_ + [*w*_*xy*_^2^]_g_)([*w*_*z*_^2^]_r_ + [*w*_*z*_^2^]_g_)^1/2^.

Fluorescent dye-labelled α-synuclein samples were diluted in phosphate buffer saline (PBS) to the nanomolar scale and measured 50 times. Fluorescence intensity auto-correlation curves were calculated and fitted to

1 $$G(\tau )=G(0)[1+\frac{T}{1-T}\exp (-\frac{\tau }{{\tau }_{T}})]\sum _{i=1}^{2}\,{f}_{i}F({\tau }_{{D}_{i}}),{\rm{where}}$$

2 $$F({\tau }_{{D}_{i}})={[1+{(\frac{\tau }{{\tau }_{{D}_{i}}})}^{\alpha }]}^{-1}\,{[1+{\{\frac{\tau }{{\tau }_{{D}_{i}}}{(\frac{{w}_{xy}}{{w}_{z}})}^{2}\}}^{\alpha }]}^{-1/2},$$

*G*(0) is the amplitude of the correlation function, *T* is the triplet-state fraction, *τ*_T_ is the triplet relaxation time of the dye, *f*_1_ is the fraction of signal from free dye, *f*_2_ is the fraction of signal from the α-synuclein construct, ${\tau }_{{D}_{1}}$ is the diffusion time of the free dye, ${\tau }_{{D}_{2}}$ is the diffusion time of the α-synuclein construct, *α* is a parameter reflecting the degree of anomalous diffusion, and *w*_*xy*_/*w*_*z*_ is the ratio of the polar and equatorial radii in the confocal volume^94,95^. Several of the fitting parameters were fixed during the measurements: *w*_*xy*_/*w*_*z*_ = 10, *τ*_T_ = 4 µs, *α* = 1, ${\tau }_{{D}_{1}}$ = 46 µs for Oregon green 488, and ${\tau }_{{D}_{1}}$ = 61 µs for Cy5. Hence only *T*, *f*_1_, and ${\tau }_{{D}_{2}}$ were treated as free fitting parameters. All fits were done using Igor Pro (Wavemetrics).

Once the diffusion time *τ*_D_ was obtained for each α-synuclein construct, the average hydrodynamic radius was calculated using^94^

3 $${R}_{h}=\frac{2{\tau }_{D}}{3\pi \beta \eta {w}_{xy}^{2}},$$

where *R*_h_ is the hydrodynamic radius, *β* is the inverse thermal energy, and *ƞ* = 0.9 cP is the PBS buffer viscosity.

### FCCS measurements and analysis

Dual-color FCCS measurements were made at different incubation times using various combinations of tandem oligomers and monomer as described in the text and listed in Table S2. For each experiment, equal volumes (125 µL each) and monomer-equivalent concentrations (536 µM, equivalent to 7.8 mg/mL) of fluorophore-labelled α-synuclein were mixed and incubated at 310 K with continuous shaking at 250 rpm to initiate the aggregation process. The protein concentrations were chosen to provide a detectable amount of aggregation within the 24-h time-frame of the measurements. 5-μL aliquots were taken from the mixture every hour, starting from the initial mixing time point, and diluted in PBS to a final concentration of ~134 pM. The diluted sample was immediately measured by FCCS at 298 K using the same microscope as for FCS studies, repeating the measurement 50 times. Each set of aggregation-dilution time-points was repeated between two and five times for each experimental condition; time-points were the same for αS-1, αS-2, and αS-4 but differed slightly in the 4–8 hr range for αS-8 owing to variations in instrument availability. The fluorescence intensity auto-correlation curves for each dye, respectively *G*_*r*_(*τ*) and *G*_*g*_(*τ*) for red and green dyes, were fit separately using Eq. 2. The cross-correlation data were fit using

4 $${G}_{rg}(\tau )={G}_{rg}(0)F({\tau }_{{D}_{rg}}),$$

where *G*_*rg*_(0) is the amplitude of the cross-correlation function. FCCS fits used the same fixed parameters as FCS, but treated *α* as a free parameter.

The number of single labelled (*N*_*r*_, *N*_*g*_) and dual labelled (*N*_*rg*_) species can be calculated from the amplitudes (*G*_*r*_, *G*_*g*_, and *G*_*rg*_) of the correlation functions. The fraction of red-labelled fluorescent species that formed aggregates was given by *N*_*rg*_/*N*_*r*_, and that of green-labelled species was given by *N*_*rg*_/*N*_*g*_. It is advantageous to present FCCS data in terms of the ratios *N*_*rg*_/*N*_*r*_ and *N*_*rg*_/*N*_*g*_, instead of the absolute numbers (*N*_*r*_, *N*_*g*_, or *N*_*rg*_), to avoid sampling errors arising from the large dilutions (~4 × 10^4^), which can cause variability in the absolute numbers of species in the confocal volume. Adjustment of the absolute volumes for the different effective confocal volumes yielded the average concentration of dual-colored aggregates, *n*_*rg*_, as well as the average concentration of all species (whether individual molecules, single-colored aggregates, or dual-colored aggregates) emitting red/green fluorescence, *n*_*r/g*_, according to:

5 $$\frac{{n}_{rg}}{{n}_{r}}=\frac{(1-{f}_{1,g}){G}_{rg}(0)}{{G}_{g}(0)}\frac{{V}_{r}}{{V}_{rg}}\,{\rm{and}}\,\frac{{n}_{rg}}{{n}_{g}}=\frac{(1-{f}_{1,r}){G}_{rg}(0)}{{G}_{r}(0)}\frac{{V}_{g}}{{V}_{rg}},$$

where *f*_1,g/r_ is the fraction of free green/red dye.

### Thioflavin T assay

ThT assays of fibril formation by monomeric α-synuclein were done under the conditions used to induce aggregation in the FCCS measurements. 10 µM ThT (SigmaAldrich) was added to 250 µL of 536 µM αS-1 in PBS, pH 7.4, shaking the solution continuously (250 rpm) at 310 K in 1.5 mL plastic microcentrifuge tubes. Time points were measured by transferring 150 µL of the solution to a 96-well plate and measuring ThT fluorescence with a PHERAstar Plus (BMG LabTech, Cary, NC, USA) microplate reader, before returning the sample to the stock tube for continued incubation. Each ThT assay was repeated three times and experimental uncertainty was reported as the standard error of the mean of the replicates.

Because of more limited sample availability, ThT assays of the tandem oligomers were performed at lower concentrations in a clear bottom, black 96-well microplate (Grenier Bio-One, Monroe, NC, USA) with a 3 mm acid-washed glass bead (SigmaAldrich) in each well. All α-synuclein constructs were present at equivalent concentrations of the monomer units: 69 µM αS-1, 34.5 µM αS-2, 17.5 µM αS-4, and 8.6 µM αS-8, each in PBS containing 40 µM ThT at a final volume of 150 µL. Proteins were incubated in the dark at 310 K with orbital shaking (250 rpm) in a Gemini XPS microplate reader (Molecular Devices, Sunnyvale, CA). Fibril content was quantified at 10-min intervals exciting ThT fluorescence at 440 nm and measuring emission at 490 nm.

### Dynamic light scattering (DLS) and zeta-potential experiments

DLS measurements of the α-synuclein constructs to determine their hydrodynamic radii and zeta potentials were performed using a Zetasizer Nano ZS instrument (Malvern Instruments Ltd., Malvern, UK) at an excitation wavelength of 633 nm and detection angle of 173°. When PBS was used as dispersant at 298 K, the refractive index was 1.338 and the viscosity 0.90 cP^96,97^. For protein, the refractive index was set to 1.45 and absorption to 0.001. Default duration time and attenuation values were used, and the sample was equilibrated for 180 s. 1.4 mg/mL of each α-synuclein construct was measured in triplicate with at least 13 runs per measurement. To avoid contamination by large aggregates, 100-nm pore size syringe filters (Whatman Anotop 10) were used to filter 1.5 mL of each protein sample directly into disposable DTS0012 cuvettes immediately prior to measurement. Zeta potential measurements for each construct were performed with the same instrument in DTS1070 folded capillary cells by applying laser Doppler velocimetry and electrophoresis to filtered samples with a concentration of 1 mg/mL. Zeta potential values were calculated using the instrument software (Zetasizer Nano ZS 7.12).

## Supplementary information

Supplementary information

## Data Availability

All data are available from the corresponding author upon reasonable request.
