# Supplementary material for: Early stages of aggregation of engineered α-synuclein monomers and oligomers in solution
Source: Sci Rep. 2019 Feb 11;9:1734. doi: 10.1038/s41598-018-37584-6 (PMC6370846; doi:10.1038/s41598-018-37584-6)
Supplement: Supplementary file 1 — Supplementary information [file 41598_2018_37584_MOESM1_ESM.pdf]

# Supplementary information:

# Early stages of aggregation of engineered α-synuclein monomers and oligomers in solution

Xi Li, Chunhua Dong, Marion Hoffmann, Craig R. Garen, Leonardo M. Cortez, Nils O. Petersen, and Michael T. Woodside

## Supplementary figures and tables

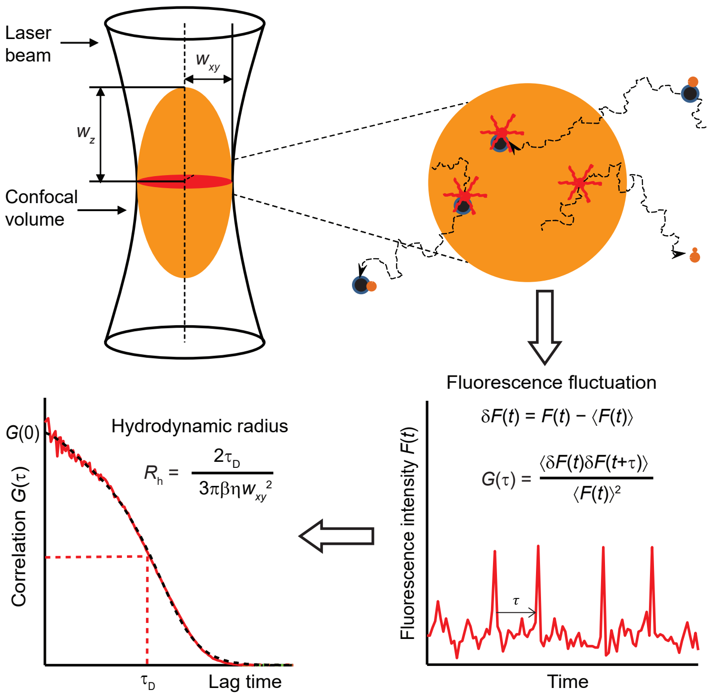


**Figure S1: FCS assay**. Schematic representation of the measurement and analysis of FCS data. Protein molecules labelled with a single dye passing through the confocal volume emit bursts of fluorescence. The decay time of the autocorrelation of the fluorescence intensity yields the hydrodynamic radius.

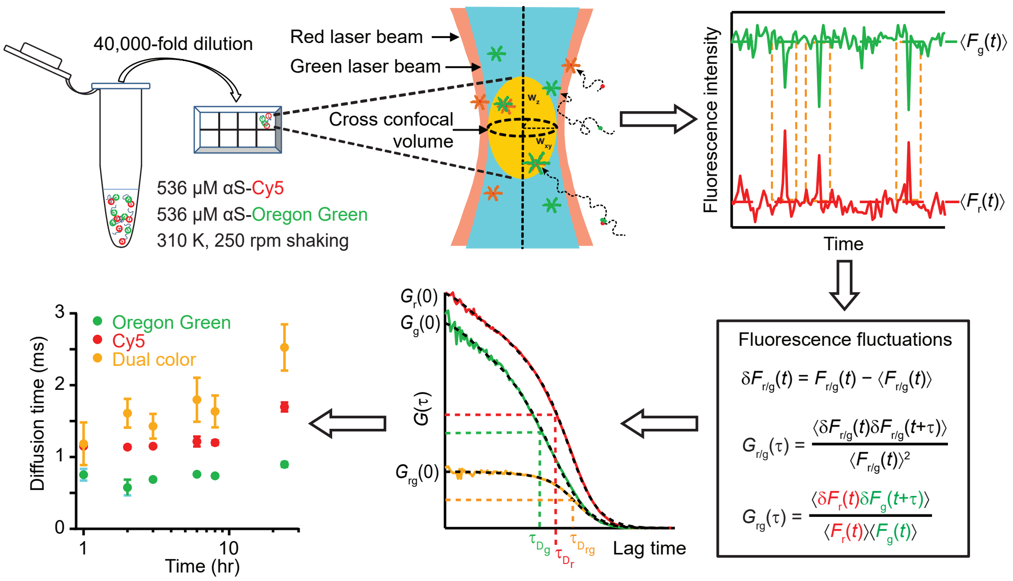


**Figure S2: FCS/FCCS assay**. Schematic representation of the measurement and analysis of FCCS data. Proteins labelled with Cy5 were incubated with other proteins labelled with Oregon green in microfuge tubes. At specific time-points, a sample was diluted for measurement. Assemblies containing red-labelled, green-labelled, or dual-labelled proteins that passed through the overlapping confocal volume (yellow) generated fluorescence fluctuations. The two auto-correlations and the cross-correlation were analyzed to determine diffusion times.

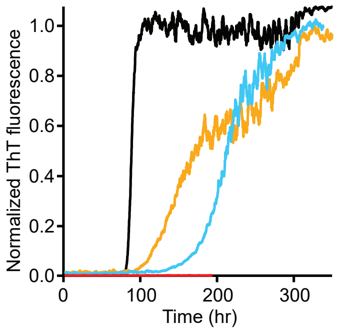


**Figure S3: ThT fluorescence assay of aggregation performed in a microplate**. Fluorescence from monomers (black), dimers (orange), tetramers (cyan), and octamers (red).

**Table S1:** The diffusion time of labeled αS is independent of protein auto-fluorescence.

| Concentration of green- labelled αS-4 (nM) | Concentration of unlabelled αS-4 (nM) | G(0) | $\tau_D$ (μs) |
|---|---|---|---|
| **34** | 10898 | 0.100±0.002 | 464±25 |
| **34** | 5449 | 0.099±0.002 | 459±21 |
| **34** | 2724 | 0.104±0.002 | 440±32 |
| **34** | 1362 | 0.092±0.002 | 450±28 |
| **34** | 681 | 0.133±0.003 | 473±34 |
| **34** | 341 | 0.104±0.002 | 456±28 |
| **34** | 170 | 0.151±0.003 | 460±27 |
| **34** | 85 | 0.129±0.004 | 476±37 |

**Table S2:** Dual-colour FCCS experiment codes.

| | **Oregon-green labelled α-synuclein** | | | |
|---|---|---|---|---|
| **Cy-5 labelled α-synuclein** | **αS-1 (536 μM)** | **αS-2 (268 μM)** | **αS-4 (134 μM)** | **αS-8 (67 μM)** |
| **αS-1 (536 μM)** | $(\alpha S\text{-}1)_r(\alpha S\text{-}1)_g$ | $(\alpha S\text{-}1)_r(\alpha S\text{-}2)_g$ | $(\alpha S\text{-}1)_r(\alpha S\text{-}4)_g$ | $(\alpha S\text{-}1)_r(\alpha S\text{-}8)_g$ |
| **αS-2 (268 μM)** | $(\alpha S\text{-}2)_r(\alpha S\text{-}1)_g$ | $(\alpha S\text{-}2)_r(\alpha S\text{-}2)_g$ | - | - |
| **αS-4 (134 μM)** | $(\alpha S\text{-}4)_r(\alpha S\text{-}1)_g$ | - | $(\alpha S\text{-}4)_r(\alpha S\text{-}4)_g$ | - |
| **αS-8 (67 μM)** | $(\alpha S\text{-}8)_r(\alpha S\text{-}1)_g$ | - | - | $(\alpha S\text{-}8)_r(\alpha S\text{-}8)_g$ |
